# Our New Friends

**Published:** 1917-05

**Authors:** 


### Our New Friends.

This month the attention of the JOURNAL'S readers is called to the advertisements of the Dae Health Laboratories, Coca-Cola, the Oaks Sanitarium, the Loyola University, and the Solar-Therapeutic Laryngoscope. Each of these firms desire your patronage. Their advertisements are interesting. Be sure and read them, and don't forget to patronize them when you desire anything in their line.

The annual meeting of alienists and neurologists will be held Monday, July 9, to Thursday, July 12, 1917, in the red room, LaSalle Hotel, Chicago, under the auspices of the Chicago Medical Society. Dr. George A. Zeller will act as chairman. The program will be mailed June 28th, with abstract of each paper. Contributors to the program are solicited. This is a society without a membership fee.

Address Secretary A. & N., Room 1218, 30 No. Michigan Avenue, Chicago.

---

Surgeon General of the Navy W. C. Braistead has inquired of Dr. William Carter, Dean of the Texas Medical College, as to the number of students who could enter the service of the United States Medical Reserve Corps at once. Dr. Carter has stated that there are a number of students who have made the grades required for the service.

---

During the past session of the Legislature the vital statistics law was so amended that it can now be enforced, and physicians are required under penalty to report all births and deaths. It is estimated that only about 69 per cent of births and deaths have been reported heretofore.

---

Dr. T. Stufit Paton, of Princeton University, acting under the direction of the Surgeon General of the Army, will establish a military hospital on the Texas border to care for mental cases among the troops. The fund provided will establish a hospital with one hundred beds.

---

If some physicians are as poor collectors as they are payers, their families must suffer from their delinquencies. To such we advise a liberal appropriation of the Golden Rule.

---

One of the first symptoms of hookworms is laziness and mental stupidity. A few of the doctors of our acquaintance should look a little out, or the hookworms will be getting them.

---

Think screening is too expensive and then blame your malaria on the climate.

